# Physical and Psychosocial Work Environmental Risk Factors for Back Injury among Healthcare Workers: Prospective Cohort Study

**DOI:** 10.3390/ijerph16224528

**Published:** 2019-11-15

**Authors:** Lars Louis Andersen, Jonas Vinstrup, Ebbe Villadsen, Kenneth Jay, Markus Due Jakobsen

**Affiliations:** National Research Centre for the Working Environment, DK-2100 Copenhagen, Denmark; jov@nrcwe.dk (J.V.); evi@nrcwe.dk (E.V.); kennethjay@mac.com (K.J.); mdj@nrcwe.dk (M.D.J.)

**Keywords:** health care sector, nurses, occupational injuries, low back pain, workplace

## Abstract

The incidence of occupational back injury in the healthcare sector remains high despite decades of efforts to reduce such injuries. This prospective cohort study investigated the risk factors for back injury during patient transfer. Healthcare workers (*n* = 2080) from 314 departments at 17 hospitals in Denmark replied to repeated questionnaires sent every 14 days for one year. Using repeated-measures binomial logistic regression, controlling for education, work, lifestyle, and health, the odds for back injury (i.e., sudden onset episodes) were modeled. On the basis of 482 back injury events, a higher number of patient transfers was an important risk factor, with odds ratio (OR) 3.58 (95% confidence interval (CI) 2.51–5.10) for 1–4 transfers per day, OR 7.60 (5.14–11.22) for 5–8 transfers per day, and OR 8.03 (5.26–12.27) for 9 or more transfers per day (reference: less than 1 per day). The lack of necessary assistive devices was a common phenomenon during back injury events, with the top four lacking devices being sliding sheets (30%), intelligent beds (19%), walking aids (18%), and ceiling lifts (13%). For the psychosocial factors, poor collaboration between and support from colleagues increased the risk for back injury, with OR 3.16 (1.85–5.39). In conclusion, reducing the physical burden in terms of number of daily patient transfers, providing the necessary assistive devices, and cultivating good collaboration between colleagues are important factors in preventing occupational back injuries among healthcare workers.

## 1. Introduction

Recent data from the Global Burden of Disease Study shows that low-back pain continues to be a leading cause of years lived with disability [1]. While low-back pain is multifactorial in origin, several work-related factors can contribute to it. Heavy lifting, frequent turns, twisting and bending of the back, are among the commonly reported work-related risk factors for low-back pain [2,3]. These are also associated with increased risk for long-term sickness absence [4,5] and early involuntary retirement from the labor market [6,7,8]. Such physical exposures are common among workers with manual material handling as well as among healthcare workers.

Healthcare workers transferring patients, e.g., nurses and nurses’ aides, are frequently experiencing back-related problems [9] often due to injuries occurring suddenly and unexpectedly during patient transfers. Several studies show an association between patient transfer and risk of back injury [10,11,12,13,14], and biomechanical studies confirm the high physical loading of the back during such work [15,16,17]. Across the European Union, healthcare workers rate their own health and safety as poorer than the rest of the working population [18], and qualitative interviews indicate that this negatively impacts quality of life and overall satisfaction with the job [19]. Altogether, back injuries can lead to long-term negative physical and psychological consequences [20]. Thus, several important reasons for preventing back injuries among healthcare workers exist.

One important initiative to prevent back injuries is ensuring the consistent use of assistive devices during patient transfer [10]. Thus, among healthcare workers in eldercare, consistent use of assistive devices is associated with a markedly decreased risk of future back injury [10]. Likewise, involving healthcare workers and their leaders in a participatory approach for improved use of assistive devices has shown to reduce the incidence of injuries to about half [21]. However, to be successful in this endeavor, a good collaboration between colleagues as well as with the leaders is probably important. An Australian study further reported that a “no lifting policy”—i.e., making it obligatory to use assistive devices during patient transfer—led to fewer back injury compensation claims [22]. However, healthcare workers often face situations where the necessary assistive devices are not readily available [23]. Knowledge about which assistive devices are commonly missing when back injuries occur may help hospitals to better plan preventive strategies.

While the majority of preventive strategies at hospitals focus on ergonomic factors, improving psychosocial factors may also be important. Thus, a recent systematic review suggests that psychosocial factors such as high demands and low job control, effort–reward imbalance, and low social support may be important risk factors for musculoskeletal disorders among healthcare workers [24]. Several studies have also highlighted the role of good leadership as important for the health status of this population [25].

The aim of this study was therefore to investigate physical and psychosocial work environmental risk factors for back injury during patient transfer among healthcare workers in hospitals. To encounter some of the methodological shortcomings of previous studies, e.g., recall bias and a long time between exposure and outcome, we used a repeated-measures design with questionnaires every 14 days during a year.

## 2. Materials and Methods

### 2.1. Study Design and Population

The design was a prospective cohort study with a baseline questionnaire in 2017 and repeated questionnaires every 14 days for one year. The baseline questionnaires were sent by e-mail to 7025 employees from 389 departments at 19 hospitals in Denmark, of which 4151 (59.1%) responded. The only inclusion criteria at the department level was that there should be some sort of patient transfer, i.e., office and administrative departments were excluded. All hospitals were public and represented two (North and Mid) of the five regions of Denmark (North, Mid, South, Zealand, Capital). Of the respondents, only groups working directly with patients (nurses, nurses aids, healthcare assistants, occupational therapists, physical therapists, midwifes, medical doctors, porters, and radiographs) were selected for further analysis (*n* = 3885). Participants received a short questionnaire every 14 days during one year after baseline. For the present analysis, we included only healthcare workers who responded to at least three of the repeated questionnaires during the 1-year follow-up period, yielding a final sample size of 2080 healthcare workers spanning 314 different departments from 17 hospitals. The mean number of repeated responses during 1-year follow-up was 12.3 (SD 7.3). Table 1 shows the baseline characteristics of the included study population (*N* = 2080 ~ 54%) as well as of the non-responders (*N* = 1805 ~ 46%) to the repeated questionnaires during follow-up.

### 2.2. Ethical Approval and Data Protection

The National Research Centre for the Working Environment has an agreement with the Danish Data Protection Agency about registering all studies in-house. According to Danish law, questionnaire- and register-based studies need neither approval from ethical and scientific committees nor informed consent [26]. All data were de-identified and analyzed anonymously.

### 2.3. Predictors

In relation to the physical work demands, the frequency of patient transfer was evaluated with the following question sent every 14 days during the one year follow-up period: *“How many patients have you transferred per day on working days during the last 14 days (if you transferred the same patient more than once per day, it counts as more patients)”* with the response options: (1) none, (2) less than one per day (e.g., 2–3 per week), (3) 1–2 per day, (4) 3–4 per day, … (12) 19–20 per day, (13) more than 20 per day [23]. An explanation was provided regarding the meaning of transfer, including some examples: *“By transfer is meant to help a patient move from one place to another or from one position to another, for example (1) from bed to wheelchair, (2) from chair to toilet chair, (3) help the patient move further up in the bed, (4) accommodate the patient in the wheelchair, (5) turn the patient, (6) situations where the patient needs to get dressed or be helped with personal hygiene”*. For the subsequent analyses, the categories were collapsed to (1) less than once per day, (2) 1–4 per day, (3) 5–8 per day, and (4) 9 or more per day.

In relation to psychosocial work factors, participants replied at baseline to questions from the Copenhagen Psychosocial Questionnaire [27] about (1) collaboration between and support from colleagues (three items), (2) influence at work (two items), and (3) recognition and support from the management (two items). Responses from the questions evaluated on each scale were averaged and normalized on a scale of 0–100 according to the test score manual (100 is best). For subsequent analyses, we defined 0–50 as ‘poor’, 50.01–75 as ‘moderate’, and 75.01–100 as ‘good’ psychosocial work environment for each of the three scales.

### 2.4. Outcome

A back injury event was evaluated with the question “*Have you injured your back during a patient transfer within the previous 14 days (think about whether the pain occurred suddenly and unexpectedly during the transfer)*”, with the response categories (1) no, (2) yes, one time, (3) yes, two times, and (4) yes, three or more times. For subsequent analyses, categories 2–4 were collapsed into ‘yes’ [10].

Those replying ‘yes’ to the occurrence of a back injury also received the following questions:

Sick leave: *“Did you have to go on sick leave due to the back injury?”* with the response options (1) no and (2) yes (indicating the number of sick leave days).

Assistive devices: “*Were the necessary assistive devices available when the back injury occurred?*” with the response options (1) no and (2) yes.

Those replying ‘no’ also received the question *“Which assistive device(s) were lacking when the back injury occurred?”* with a 16-item multiple-choice list of assistive devices.

In short, this list included the vast majority of assistive devices used during patient transfer, spanning from common friction-reducing devices (e.g., sliding sheets, sliding boards, and masterturners) characterized by a manual approach to horizontal transfer and/or repositioning in bed, to devices utilized when moving the patient from one room to another (e.g., walking aids, wheelchairs, gait belts, and stand-assist lifts). More technologically advanced devices (e.g., ceiling lifts, intelligent beds, and electric versions of the masterturner) most commonly used when transferring old, frail, and/or bariatric patients within the room (e.g., from bed to chair) were also included.

### 2.5. Control Variables

To control for possible confounding, we included basic variables about work, health, and lifestyle from the baseline questionnaire. Basic variables: age (continuous variable) and sex (female, male). Work-related factors beside the predictor variables: healthcare specific education (categorical variable, e.g., nurse, medical doctor, physical therapist, etc.), seniority (years working as healthcare worker, continuous variable). Health: mental health from SF-36 (continuous variable) [28], low-back pain intensity during the previous month (continuous variable, 0–10) [29]. Lifestyle: body mass index (BMI = weight/height^2^, continuous variable), smoking status (daily smoker, not daily smoker, ex-smoker, non-smoker), leisure physical activity (4 categories, from sedentary to a very high level of leisure physical activity) [30]. From the repeated short questionnaires sent every 14 days, the analysis was controlled for the number of working days during the last 14 days (continuous variable), i.e., in the same period as the predictor variable ‘number of daily patient transfers’ and for previous back injury using the reply provided 14 days earlier.

### 2.6. Statistical Analysis

Using repeated-measures binomial logistic regression with random effects modeling, we estimated the risk for back injury events during follow-up. The dataset was re-arranged for the predictor variable (number of patient transfers) to always come 14 days before the outcome variable, and the control variable of previous back injury to come 14 days before the predictor variable. This allowed an analysis of the prospective short-term association between exposure (patient transfer) and the risk of back injury 14 days later, controlling for previous back injury in the previous 14 days. The analysis was mutually controlled for the number of patient transfers and the psychosocial variables (i.e. included in the same model), as well as for the variables previously mentioned (Section 2.5. Control variables). Further, it was adjusted for clustering at the department level using the ‘random’ statement of PROC GLIMMIX (SAS v9.2). Using the ‘random _residual_’ statement, the analysis also took into account the fact that each participant provided several repeated measures during follow-up. The containment method was used to obtain degrees of freedom. The main results are provided as odds ratios (OR) and 95% confidence intervals (95% CI). Other descriptive statistics are provided as means (SD) and prevalence (percentage, %).

## 3. Results

Table 1 shows that, at baseline, the mean age of the responders to the repeated questionnaire was 48 years, while that of the non-responders was 45 years and the majority of the healthcare workers were women. The majority of workers had daily patient transfers. Mental health was on average normal (>80), and the intensity of low-back pain was about 2 in both responders and non-responders. During the last year prior to baseline, 10.2% and 13.0% of the responders and non-responders, respectively, had experienced at least one back injury (i.e., sudden onset episode) during patient transfer. For the lifestyle factors, BMI was on average about 25, there were only few smokers, and the majority (about 60%) performed light physical activity during leisure.

During the one-year follow-up period, there were 482 reported back injury events. The unadjusted incidence of back injuries during the last 14 days was 0.3%, 2.4%, 5.4%, and 7.0% among those with less than 1, 1–4, 5–8, and 9 or more patient transfers per day, respectively (not shown in the tables). Of the back-injury events, 7.8% led to sickness absence of 1 day or more, with an average of 3.8 days [SD 4.0] (not shown in the tables).

Table 2 shows the fully adjusted analysis between number of daily patients transfer during the last 14 days and the risk for back injury, as well as between the psychosocial work environmental factors at baseline and the risk for back injury. A higher number of patient transfers was—in an exposure–response fashion—an important risk factor, with OR 3.58 (95% CI 2.51–5.10) for 1–4 transfers per day, OR 7.60 (5.14–11.22) for 5–8 transfers per day, and OR 8.03 (5.26–12.27) for 9 or more transfers per day (reference: less than 1 per day). In a trend test, i.e., using the number of patient transfers as a continuous variable, it was also highly significant in relation to back injury events (*p* < 0.001). For the psychosocial factors, poor collaboration between and support from colleagues increased the risk, with OR 3.16 (1.85–5.39). In a trend test, i.e., using collaboration between and support from colleagues as a continuous variable, it was also significant (*p* < 0.01). Influence at work as well as recognition and support from management were not significant risk factors for back injury in the present analysis.

In 26.4% of the back injury events during patient transfer, the healthcare workers reported that one or more of the necessary assistive devices were not available. Table 3 shows which assistive devices were most commonly lacking when a back injury event occurred. The top four lacking devices were sliding sheets (30%), intelligent beds (19%), walking aids (18%), and ceiling lifts (13%).

## 4. Discussion

This study investigated physical and psychosocial work environmental risk factors for back injury during patient transfer among healthcare workers at hospitals. The main finding is that a higher number of patient transfers as well as poor collaboration between and support from colleagues appeared as risk factors for back injury. In the specific situations where back injuries occurred, the healthcare workers often lacked the necessary assistive devices, most commonly sliding sheets, intelligent beds, walking aids, and ceiling lifts.

The number of daily patient transfers was—in an exposure–response fashion—a risk factor for sustaining a back injury during patient transfer. This confirms previous findings in the eldercare sector [10], although the odds ratios were much higher in the present study. A difference between this and our study is that the previous study only had a 1-year follow-up questionnaire and did not have repeated measures. Because exposure and injury are often temporally related—i.e., an unexpected high mechanical load may cause a sudden injury—using repeated questionnaires increases the chance of finding an association between exposure and risk of injury two weeks later. However, an injury may also be preceded by accumulated exposure that ultimately leads to the injury event characterized by a sudden and unexpected back pain occurring during patient transfer. To account for exposure that may have led to a level of discomfort or pain, but not (yet) resulted in an actual injury, we controlled for low-back pain intensity at baseline. Likewise, the analysis was controlled for previous back injury, which is a strong predictor of future injury [31]. Lastly, we controlled for mental health and lifestyle factors, which have also been linked to the development of low-back pain [32,33,34].

Aside from physical exposure, this study also evaluated the availability of necessary assistive devices when a back injury event occurred. Equipment availability constitutes one of the most cited factors influencing safe patient transfer scenarios [35], and—perhaps most importantly—nurses themselves perceive this as the most effective component in decreasing the frequency of lifting-related accidents [36]. In the present analysis, we report that the most commonly lacking assistive devices were, in descending order, sliding sheets, intelligent beds, walking aids, and ceiling lifts. Considering that not only the general use of assistive devices decreases the risk of back injury [10], but also specific groups of assistive devices are associated with lower physical load than others (e.g., ceiling lifts and intelligent beds) (Vinstrup 2019 under review), the fact that healthcare workers consistently report lack of equipment as a reason to engage in unsafe patient transfers remains highly problematic. Further, considering the low cost of the sliding sheet (i.e., a friction-reducing sheet placed underneath the patient), it seems prudent to make sure that this specific assistive device is readily available in all departments.

Biomechanical laboratory studies have shown that muscular load during patient transfer is lower when using the ceiling lift than when using the traditional floor lift [37]. However, another study showed equally reduced compression forces of the low-back using the ceiling and floor lifts [38]. Similarly, slings also reduce back compression forces albeit not as effectively as lifts [38], whereas utilizing the sliding sheet has been shown to reduce the biomechanical compression force on the low back [16]. In contrast, two recent systematic reviews of longitudinal intervention studies found limited evidence for preventive interventions with assistive devices to reduce musculoskeletal pain and injuries among healthcare workers [39,40,41], indicating that low physical loads and the availability of assistive devices are only a part of the puzzle. However, adequate implementation of the intervention or the description hereof is often lacking in intervention studies, and whether the lack of preventive effect reported in systematic reviews is due to efficacy failure or implementation failure remains uncertain. While performing multiple randomized controlled trials is unfeasible and costly, well-controlled prospective cohort studies can provide an alternative approach to shed light on the association between work-related factors of patient transfer and the risk for back injury.

Regarding the psychosocial work factors, we found that poor collaboration between and support from colleagues was a risk factor for back injury. This is in line with a review showing that poor social support may be a risk factor for musculoskeletal disorders among healthcare workers [24]. Thus, fostering good collaboration between colleagues and mutual support seems to be important for the local working environment. There may be several explanations for this finding: First, mutual supporting in busy periods may indirectly reduce individual physical workload as well as individual distress. Second, by solving the tasks together in teams, the individual healthcare worker may reduce the physical workload when dealing with ‘heavy’ and relatively immobile patients. Third, it may be easier to find and use appropriate assistive devices when good collaboration between colleagues exists. Thus, there may be several direct and indirect reasons for the importance of good collaboration between colleagues in the prevention of back injuries.

Several studies have highlighted good leadership as important for the health of healthcare workers [25]. Surprisingly, we did not find a significant influence of recognition and support from the management for the risk of back injury. Nevertheless, it should be remembered that the management can have an important indirect role by securing a good overall work environment that facilitates collaboration between and support from other colleagues when needed. In addition, we did not find a significant association between influence at work and risk of back injury, although we expected that healthcare workers with a higher degree of influence at work would be able to better plan their work to avoid unnecessary high workloads and injuries. Nevertheless, previous studies have reported inconsistent results regarding the importance of influence at work in relation to health outcomes [42,43,44].

### Strengths and Limitations

The present study has both strengths and limitations. A strength is the repeated-measures design, which increased the statistical power and allowed the investigation of the temporal associations between exposure and risk of injury. Furthermore, recall bias was likely very limited, as the questionnaires were sent out every 14 days. By contrast, many studies use retrospective reporting of up to one year of exposure or outcome, which makes recall bias much more likely. A limitation of such design is the difficulty in getting people to reply repeatedly over a year. Thus, 46% of the baseline population chose not to participate in the repeated questionnaires during 1-year follow-up. However, on the basis of the baseline characteristics (Table 1), there were only minor differences between the responders and the non-responders. Furthermore, controlling for a number of confounders increased the validity of the findings.

Regarding the sample size, we previously found strong exposure–response associations between manual lifting and risk of acute back pain using a repeated-measures design with less than 100 workers in the supermarket sector [45]. However, to increase the generalizability of the present study, we aimed to include as many healthcare workers from as many hospitals in Denmark as possible. With a final sample of 2080 healthcare workers spanning 314 different departments from 17 different hospitals, the results are likely generalizable to all hospitals in Denmark, although only two of the five regions of Denmark were represented.

## 5. Conclusions

In conclusion, reducing the physical burden in terms of number of daily patient transfers, providing the necessary assistive devices, and cultivating good collaboration between colleagues are important for preventing occupational back injuries among healthcare workers.

## Figures and Tables

**Table 1:** Demographics, work, health, and lifestyle at baseline. Results are either mean (SD) or prevalence as percentage (%) of the study population. BMI: body mass index.

Variable	Study Population	Non-Responders
*N*	2080	1805
Age (mean)	48.2 (11.1)	44.5 (11.5)
Gender (% women)	87.1%	86.4%
Seniority, years (mean)	17.9 (11.7)	15.0 (11.4)
**NUMBER OF DAILY PATIENT TRANSFERS (%)**		
Less than 1	39.3%	34.3%
1–4	28.4%	30.9%
5–8	17.9 %	18.8%
9 or more	14.4%	16.1%
**PSYCHOSOCIAL WORK FACTORS (0–100, where 100 is best)**		
Collaboration between and support from colleagues	80.0 (13.9)	78.0 (14.6)
Influence at work	73.5 (17.6)	70.0 (18.8)
Recognition and support from management	69.2 (20.9)	64.3 (22.6)
**HEALTH FACTORS**		
Mental health (0–100, where 100 is best)	82.2 (13.4)	80.3 (14.2)
Low-back pain intensity (0–10)	2.4 (2.6)	2.3 (2.5)
Previous back injury (%)	10.2%	13.0%
**LIFESTYLE FACTORS**		
Smoking (% yes)	8.1%	10.6%
BMI (mean)	25.4 (4.8)	24.8 (4.7)
Leisure physical activity (%)		
1. Sedentary	6.5%	7.4%
2. Light activities for at least 4 h per week	61.7%	58.4%
3. Physical exercise or other strenuous activities for at least 4 h per week	28.9%	29.9%
4. Hard physical exercise and competitions on a regular basis	3.0%	4.3%

**Table 2:** Odds ratios and 95% confidence intervals for the risk of back injury events during the 1-year follow-up period. All predictor variables are mutually adjusted. Statistically significant findings are marked in bold.

Predictor Variable	*n*	%	OR (95% CI) ^a^
**Number of daily patient transfers ^b^**			
Less than 1	13543	53.3	1
1–4	7223	28.4	**3.58 (2.51–5.10)**
5–8	2575	10.1	**7.60 (5.14–11.22)**
9 or more	2061	8.1	**8.03 (5.26–12.27)**
**Collaboration between and support from colleagues ^c^**			
Good	1051	51.2	1
Moderate	917	44.7	1.09 (0.82–1.43)
Poor	85	4.1	**3.16 (1.85–5.39)**
**Influence at work ^c^**			
Good	606	29.5	1
Moderate	1089	53.0	1.00 (0.73–1.36)
Poor	358	17.4	1.20 (0.81–1.79)
**Recognition and support from management ^c^**			
Good	572	27.9	1
Moderate	928	45.2	1.27 (0.91–1.78)
Poor	553	26.9	1.01 (0.68–1.51)

^a^ adjusted for gender, age, number of working days in the last 14 days, education, seniority, previous back injury, mental health, low-back pain intensity, body mass index, smoking status and leisure physical activity; ^b^ repeated measures every 14 days during the year, i.e., accumulated n; ^c^ measured at baseline; bold: Statistically significant, *p* < 0.001.

**Table 3:** Prevalence as percentage (%) of necessary assistive devices that were lacking in relation to back injury events among those who stated that one or more assistive devices were lacking.

Assistive Device that was Lacking	Percentage of Back Injury Cases
Sliding sheet	29.6%
Intelligent bed	19.0%
Walking aids	17.6%
Ceiling lift	12.7%
Floor lift	12.0%
Hospital bed	12.0%
Masterturner, electric	12.0%
Sling	11.3%
Wheelchair	9.9%
Masterturner	9.9%
Stand-assist lift	8.5%
Sliding boards	7.8%
Standing lift	7.8%
Gait belt	5.6%
Toilet-chair, electric	4.9%
Toilet-chair	4.2%

## Data Sharing Statement

Researchers interested in using the data should contact the project leader Lars L. Andersen.
